# Improving Prediction Accuracy of “Central Line-Associated Blood Stream Infections” Using Data Mining Models

**DOI:** 10.1155/2017/3292849

**Published:** 2017-09-20

**Authors:** Amin Y. Noaman, Farrukh Nadeem, Abdul Hamid M. Ragab, Arwa Jamjoom, Nabeela Al-Abdullah, Mahreen Nasir, Anser G. Ali

**Affiliations:** ^1^Department of Computer Science, Faculty of Computing and Information Technology, King Abdulaziz University, Jeddah, Saudi Arabia; ^2^Department of Information Systems, Faculty of Computing and Information Technology, King Abdulaziz University, Jeddah, Saudi Arabia; ^3^Clinical Epidemiology & Infection Control, Faculty of Nursing, King Abdulaziz University, Jeddah, Saudi Arabia; ^4^Department of Computer Science and Software Engineering, University of Hail, Hail, Saudi Arabia

## Abstract

Prediction of nosocomial infections among patients is an important part of clinical surveillance programs to enable the related personnel to take preventive actions in advance. Designing a clinical surveillance program with capability of predicting nosocomial infections is a challenging task due to several reasons, including high dimensionality of medical data, heterogenous data representation, and special knowledge required to extract patterns for prediction. In this paper, we present details of six data mining methods implemented using cross industry standard process for data mining to predict central line-associated blood stream infections. For our study, we selected datasets of healthcare-associated infections from US National Healthcare Safety Network and consumer survey data from Hospital Consumer Assessment of Healthcare Providers and Systems. Our experiments show that central line-associated blood stream infections (CLABSIs) can be successfully predicted using AdaBoost method with an accuracy up to 89.7%. This will help in implementing effective clinical surveillance programs for infection control, as well as improving the accuracy detection of CLABSIs. Also, this reduces patients' hospital stay cost and maintains patients' safety.

## 1. Introduction

 Surveillance of antibiotic resistance and nosocomial infections is a major function in a hospital infection control program [1, 2]. Nosocomial surveillance was based on ward visits, medical charts reviews, and paper based reports [3, 4]. For analysis purposes, hand-written data was assembled or manually entered into the databases. These methods tended to be time-consuming, required more personnel, and yielded inefficient results. This gave rise to the design and utilization of various computer applications and surveillance systems for infection control. Design and implementation of a surveillance system undergo various phases and have to follow several guidelines in order to produce effective results. One of the main challenges faced in healthcare systems is the effective prediction of nosocomial infections (also called healthcare-associated infections or hospital-acquired infections (HAI)). These are the infections that are not present at the time of a patient's admission at a hospital, rather these are caused as a result of some procedure used to treat the patient's illness during his stay at hospital. Common examples of such infections may include central line-associated blood stream infection (CLABSI), surgical site infections (SSI), Urinary Tract Infection (UTI), and Methicillin-resistant* Staphylococcus aureus* (MRSA) infection. These infections increase patient's stay at hospital causing additional burden on the healthcare management by occupying more resources and increasing cost of care [5]. Therefore, there is a need to have an efficient clinical surveillance program (CSP), which will investigate the patients' data and provide useful insights for predicting possibility of such infections.

In this study, we developed DM methods to predict central line-associated blood stream infections (CLABSI). We selected HAI datasets of US hospitals and integrated them with consumer experiences of hospitals. Development of a CSP using DM requires several steps that have to be designed specifically to meet the given needs [6, 7]. We followed the standard CRISP-DM methodology [8] and developed DM prediction models that can predict possibility of CLABSI with up to 89.7% accuracy.

The rest of the paper is arranged as follows. We review the relevant literature in Section 1.1. Hospital-acquired infections DM related materials and methods are explained in Section 2.  Section 3 presents major challenges in implementing a DM process for a CSP.  Section 4 discusses in detail our DM efforts for constructing a predictive model for a CSP.  Section 5 is the conclusion.

### 1.1. State of the Art

*Data mining (DM)* is a process of exploring and analyzing large datasets to discover novel interesting patterns that can be used to solve some problems or avail future opportunities. Various statistical and analytical techniques are used in DM methods to select, analyze, and transform huge datasets into useful patterns. DM has many applications and is widely used in market basket analysis [10], customer relationship management [11], and credit card fraud detection [12] and for detecting anomalies in data streams [13].

Among the various applications of DM, its use in healthcare is no exception. It has proved to be very beneficial for analyzing medical datasets and extracting powerful patterns [14–17]. The reason behind its popularity is that the data produced by healthcare organizations is voluminous and very complex in nature. Use of DM in a CSP enhances surveillance by investigating nosocomial infections among patients. Such information helps the clinicians and medical practitioners to make important decisions about patient's health, for example, identifying diseases having common symptoms and grouping the patients showing similarity in having particular disease type. Additionally, it can provide several other benefits including effective health policy making, development of surveillance systems for the prediction of infections, and determining the length of patient's stay in the hospital, to name a few. Much work done to address the challenge of effective prediction of nosocomial infections. Several techniques using rule-based approaches [18–20], Bayesian Networks [10, 21], Ontologies [22], landmark competing risk prediction models [23], statistical models [24–27], case based reasoning [28, 29], and others [30–33] have been suggested and implemented in the past. The problem of identifying new, unanticipated, and useful patterns in public health surveillance and hospital infection control data is discussed in [19]. The authors used association rules to serve the purpose. They emphasized the use of data mining methods for automated discovery of useful patterns from public health surveillance and hospital infection control data.

A study in [34] has discussed the development of an intelligent system called MONI (Monitoring of Nosocomial Infections) for detection and surveillance of healthcare-associated infections in intensive care units. The authors used methods from artificial intelligence, fuzzy sets, and medical knowledge engineering. A clinical data warehouse for controlling nosocomial infections is outlined in [34]. It included identification of data sources, acquisition of data, and its modeling and evaluation. The system was efficient and economical in terms of money. It has many good features including reducing the manual reviews and providing easy access to reports on the use of antimicrobials and trends in their resistance. Additionally, as compared to the manual process, it enhances the efficiency in identifying new positive cultures in patients.

Surveillance systems based on service oriented architecture (SOA) were proposed in [35, 36]. Tseng et al. [35] developed a web-based surveillance system for HAIs using SOA to deal with compound electric health records (EHR). The system proved to be efficient for HAI detection and assists physicians and healthcare providers in their daily work. It has also enhanced quality of medical services and patient safety. One drawback of the proposed system is that the EHR considers only body temperature as a symptom. These approaches are sometimes unable to detect other cases because of lack of information about other factors. Another SOA based HAI surveillance information system to integrate surveillance data from multiple information systems is presented in [37]. The authors developed algorithms based on discriminant analysis to detect suspicious cases. Their proposed architecture is flexible to handle different levels of HAI surveillance workflows.

Data mining has also been used in other areas of healthcare. Santos et al. [38] applied several data mining techniques including decision trees, Naive Bayesian classification, rule-based classifier, and *k* nearest neighbor classification on hospital data to build prediction models for antibiotic sensitivity. The study showed that use of different feature selection methods like wrapper-based and filter-based methods (for the analysis of important features) was helpful in predicting the pathogens sensitivity to antibiotics. An automated system based on data mining methods to analyze diagnostic reports of brain tumors is discussed in [39]. This work highlighted the significance of ontologies in data mining based automated systems. The proposed system showed sufficient decrease in development time. The authors in [22] developed a scoring system for the prediction of HAI. The system turned out to be useful to efficiently identify the patients with high risk for having HAIs during their stay in the hospital. The authors in [15] proposed a framework to address development, assessment, and application of data mining models in clinical medicine. The study showed that the decision trees are more effective as compared to Naive Bayes.

## 2. Hospital-Acquired Infections DM Related Materials and Methods

The authors in [14] described a Data Mining Surveillance System. This work classified the surveillance techniques into two categories: Machine Learning and Descriptive Mining. The proposed system used frequent set and association rule analysis to automatically construct patterns of statistical and clinical interest from laboratory medicine and patient movement data. The use of association rule method for automatic identification of novel and potentially useful patterns in HAI surveillance data for infection control is mentioned in [18]. The authors designed a method based on association rule method and extending work by Brossette and Hymel Jr. [14] for automatic detection of temporal trends. They emphasized mining for low-support, low-confidence rules for detecting unexpected outbreaks caused by a small number of cases. The main features of the system included efficient data preprocessing to reduce the search space. Additionally, it assisted the infection control practitioners by using rule templates to filter out less or not important rules. Use of association rules for development of a decision support system for an early and accurate detection of Surgical Site Infection (SSI) is presented in [47]. The system is able to detect SSI in advance and can improve patient safety by early investigation. The authors in [36] described a knowledge-based system for microbiological laboratory. Classification and rule-based approach are used for data validation and bacterial infections monitoring. A framework for process mining in critical care units is proposed in [48]. The authors extended CRISP-DM model to include temporal and multidimensional aspect. The presented system could discover knowledge of new conditions onset pathophysiologies using temporal data mining of physiological data streams. The study in [11] presented a model to integrate data from different healthcare facilities. The study extracted interesting and valid patterns to predict HAI using regression and classification. This web-based framework could successfully identify outbreaks in antibiotic resistance by analyzing hospital data.

Dynamic Bayesian Network is used in [13] to forecast the possibility of getting a nosocomial infections on daily basis. The proposed methods estimated the possibility of infections considering patient state described in terms of static as well as temporal data. The static data included entry and exit dates, gender, age, weight, and antecedents. The temporal data included daily control measurements (e.g., infectious examinations, urinary probe, intubation, catch of antibiotics, and the Central Venous Catheter). To predict the inpatient length of stay, the authors in [49] used Naive Bayesian inference models. The authors demonstrated that these models can be very helpful to enhance the classification accuracy as they are capable of handling missing data in an efficient way. Another study grouped patients according to their length of stay by using hierarchical clustering approach explained in [50].

*In this paper, we compared six data mining methods for predicting CLABSI, to select the most efficient method, using datasets of US hospitals and integrated them with consumer experiences of hospitals, as described in detail in the next sections.*

## 3. Challenges in Implementing Data Mining Process for Clinical Surveillance Program

Healthcare data has some characteristics that make it distinctly challenging to mine using automatic methods as briefly described here.

### 3.1. High Dimensionality Data

Dimensionality of a dataset represents number of attributes in the dataset. The healthcare datasets consist of attributes that represent observations or features relevant to different domains and thus have high dimensionality. It means that a variety of different data elements exists, each of which may represent a dimension whose value can vary. A patient's record might consist of 50–100 or even more different types of attributes. This high number of attributes increases the possibility of shared coincidental patterns. In addition to data exploration, the quality verification of such large datasets is also not an easy job. Constructing a model from such a large number of attributes lacks in accuracy. So, there is a need to use appropriate techniques to reduce the number of attributes to improve model accuracy [6, 7, 15]. Such techniques (referred to as data reduction techniques) identify the attributes which are more relevant to the outcome than other attributes. The attributes with lesser relevance (or no relevance) are eliminated from the main dataset and the actual model is constructed from the more relevant attributes only.

The data reduction is very sensitive task and must be performed very carefully considering the following factors:

1. Analyzing the data in order to make sure its attributes are significant and relevant
2. Determining the priority of attributes having high relevance for prediction
3. Considering the relationship between the attributes and the outcome
4. Finding the relationships among various attributes

### 3.2. Heterogenous Data Representations

Medical data is gathered from different sources resulting in different representations like images, discrete values with multiple scales, text descriptions, and paper based reports or even data warehouses. To apply DM, the data needs to be preprocessed and transformed to a structured representation in order to make useful analysis.

### 3.3. Human Based Interpretation

Patient diagnoses are usually human based, which are interpreted on the basis of various observations and objective data values. A conflict may arise when different individuals perform interpretations. Another challenge is that such observations are represented in textual form that need to be processed in a form applicable during DM.

### 3.4. Data Inconsistency

Clinical data may be inconsistent or conflicting for various reasons. One of them is that patients who have the same conditions may have different types and timing of observations. These qualities add noise and false patterns that increase the difficulty of identifying real patterns of interest. The quality of data is also an important requirement for mining purposes. The data may have records with missing values of some attributes. If such values are not calculated by using appropriate means or incomplete records are not removed, they can cause poor analytical results [6, 7]. A number of methods can be used to replace the missing values by using measures like mean and median [51].

### 3.5. Data Privacy Issues

The privacy and protection of clinical data are of primary concern. It requires that the confidentiality of patients must be ensured during DM [6]. Various controversies and confusion exist related to the ownership and usage of patient records that make this analysis complicated [7]. The data privacy issues do not prevent the use of DM in clinical domain but require extensive efforts to ensure privacy during data processing.

## 4. DM for a Clinical Surveillance Program

The goal of a CSP is to monitor patients' conditions for nosocomial infections and alarm the health practitioners about possible infections so that the precautionary measures can be taken to avoid the possibility of infections. The objective of using DM for CSP is to develop a predictive model that can predict (with reasonable accuracy) the possibility of CLABSI to a patient. DM is getting popularity in the healthcare field due to several reasons including availability of huge amounts of data to be processed and organizations' needs to make decisions on the basis of financial and clinical data and to produce equally useful information to all stakeholders in the healthcare industry [52]. There are various applications of DM in healthcare like evaluation of effectiveness of different treatments, healthcare management, and relationships between patients and care providers, to name a few. The development of the predictive model using DM methods for a CSP undergoes several different phases from its beginning to its end. In current study,* we followed cross industry standard process for DM (CRISP-DM) [8], which includes six phases as described in the following subsections.*

### 4.1. Business Understanding

Business/problem understanding is the initial phase towards implementing DM process for a clinical surveillance program. Mining of medical data requires specific medical knowledge as well as knowledge of DM technology, in order to predict risk of nosocomial infections (or hospital-acquired infections). Particularly, CLABSI in this study will using the knowledge of previous patients [13]. The case of the nosocomial infections is that people visit hospital for medical treatment of different diseases. During their treatment, they might have to get admitted to the hospital, where they might get some infections during their initial treatment. These infections are referred to as nosocomial infections. The patients suffering from nosocomial infections stay at hospital for a longer duration and use extra resources. Each such patient cost a hospital about SR 5000/per day. If incidents of such infections can be predicted in advance, effective measures can be taken to avoid the infections and thus save valuable resources for other patients. The goal of this DM study is to predict possibility of nosocomial infections at a hospital in advance.

### 4.2. Data Understanding

Data understanding phase consists of the following main tasks:

- Data collection
- Data exploration
- Data selection
- Data integration

**Figure 1:**
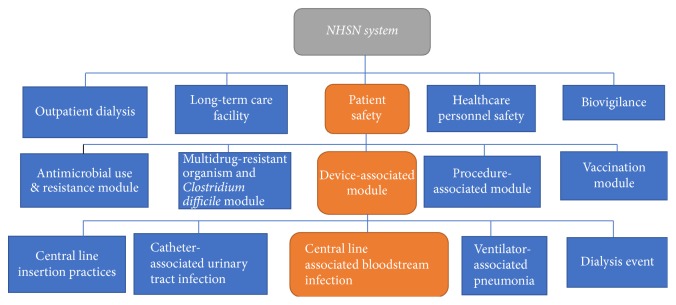
Partial structure of NHSN system depicting focused area of current study [9].

The main DM process starts from data collection. Collection of data is driven by requirement specifications in business understanding phase [53]. For current study, we selected HAI dataset from* National Healthcare Safety Network* (NHSN) [54] of* Centers for Disease Control and Prevention's* (CDC) [55] as primary dataset (represented as NHSN: HAI). CDC is the leading national public health institute of the United States. The NHSN is an Internet-based surveillance system that integrates patient healthcare personnel and safety surveillance systems at CDC. NHSN provides data needed to identify problem areas, measure progress of prevention efforts, and ultimately eliminate healthcare-associated infections. NHSN is US most widely used healthcare-associated infection tracking system. To highlight our focused area in NHSN, Figure 1 shows a partial structure of NHSN components.

The NHSN mainly consists of five major components shown in layer 1 from the top of Figure 1. Our work falls under* patient safety* component marked with orange rectangle in layer 1 of the figure. The* patient safety* component has five modules (as shown in layer 2 of Figure 1) that target five different areas. In these modules, our work falls under* device associated module* (marked with orange rectangles in layer 2 of the Figure). The* device associated module* covers five different categories shown in layer 3. This study focuses on CLABSI marked with orange rectangle in layer 3 of Figure 1. The NHSN: HAI dataset was complemented in the year 2015 with consumer assessment dataset for hospitals from* Hospital Consumer Assessment of Healthcare Providers and Systems* (HCAHPS) [56]. It was a project by Centers for Medicare and Medicaid Services. This dataset comprised 255,091 records for 4,638 hospitals.

The data miner needs to understand the data items in the dataset with their interpretation and their possible relations. This is the objective of second task of* data exploration*. In exploration of NHSN: HAI dataset, we found that this data comprises 48 attributes for the following 4 different infections:

- Central line-associated blood stream infection (CLABSI)
- Surgical site infection (SSI)
- Urinary Tract Infection (UTI)
- Methicillin-resistant* Staphylococcus aureus* (MRSA) infection

Doctor-patient communication is also a major component of the process of healthcare, as explained in [57], leading to better, safer healthcare [58].

The HCAHPS dataset is comprised of answers of 27 questions about their recent hospital stay. Among these questions, there were 18 core questions about critical aspects of their hospital experiences. These questions covered the following main aspects [56]:

- Communication with nurses (3 questions)
- Communication with doctors (3 questions)
- The responsiveness of hospital staff (2 questions)
- The cleanliness and quietness of the hospital environment (2 questions)
- Pain management (2 questions)
- Communication about medicines (2 questions)
- Discharge information (2 questions)
- Overall rating of hospital (1 question)
- Willingness to recommend the hospital to others (1 question)

A DM process must be specific to address a well-defined task for predicting the possibility of CLABSI in our case. Therefore it is very critical to identify the target data from NHSN: HAI and HCAHPS datasets. A careful selection of the variables and relevant data can lead to quickly discover the useful patterns during DM. These activities are accomplished under* data selection* task which is the third task of* data understanding* phase. The selection of data was accomplished in two rounds. In the first round, the attributes specific to the focus area were selected from the available datasets. From NHSN: HAI dataset, the attributes specific to CLABSI only were selected in round 1. These attributes are as follows:

- CLABSI: number of procedures
- CLABSI: central line days
- CLABSI: observed cases
- CLABSI: predicted cases
- CLABSI: lower confidence limit
- CLABSI: upper confidence limit
- CLABSI in ICUs only
- CLABSI in ICUs and selected wards only

Similarly, from HCAHPS datasets, answers to four questions were selected in round 1. These questions targeted 4 aspects of patients' experience including the following:

- Communication with doctors (1 question)
- Communication with nurses (1 question)
- Responsiveness of hospital staff (1 question)
- Cleanliness of hospital environment (1 question)

There were total 12 different responses of these questions (three for each question). The questions and their corresponding responses are as follows [9, 56].

*Question  1*. “How often did doctors communicate well with patients (composite measure)?”

The corresponding responses were as follows:

- “Patients who reported that their doctors* always* communicated well”
- “Patients who reported that their doctors* usually* communicated well”
- “Patients who reported that their doctors* sometimes* or* never* communicated well”

*Question  2*. “How often did nurses communicate well with patients (composite measure)?”

The corresponding responses were as follows:

- “Patients who reported that their nurses* always* communicated well”
- “Patients who reported that their nurses* usually* communicated well”
- “Patients who reported that their nurses* sometimes* or* never* communicated well”

*Question  3*. “How often did patients receive help quickly from hospital staff (composite measure)?”

The corresponding responses were as follows:

- “Patients who reported that they* always* received help as soon as they wanted”
- “Patients who reported that they* usually* received help as soon as they wanted”
- “Patients who reported that they* sometimes* or* never* received help as soon as they wanted”

*Question  4*. “How often were patients' rooms and bathrooms kept clean (individual measure)?”

The corresponding responses were as follows:

- “Patients who reported that their room and bathroom were* always* clean”
- “Patients who reported that their room and bathroom were* usually* clean”
- “Patients who reported that their room and bathroom were* sometimes* or* never* clean”

In the second round, the attributes of the first round were further screened out on the basis of the related work and their suitability to predict the future cases. The following 4 attributes were selected from NHSN: HAI dataset in round 2:

- CLABSI: number of procedures
- CLABSI: central line days
- CLABSI: observed cases
- CLABSI in ICUs only

Likewise, one answer for each of the HCAHPS questions was selected in round 2:

- “Patients who reported that their doctors* sometimes* or* never* communicated well”
- “Patients who reported that their nurses* sometimes* or* never* communicated well”
- “Patients who reported that they* sometimes* or* never* received help as soon as they wanted”
- “Patients who reported that their room and bathroom were* sometimes* or* never* clean”

**Figure 2:**
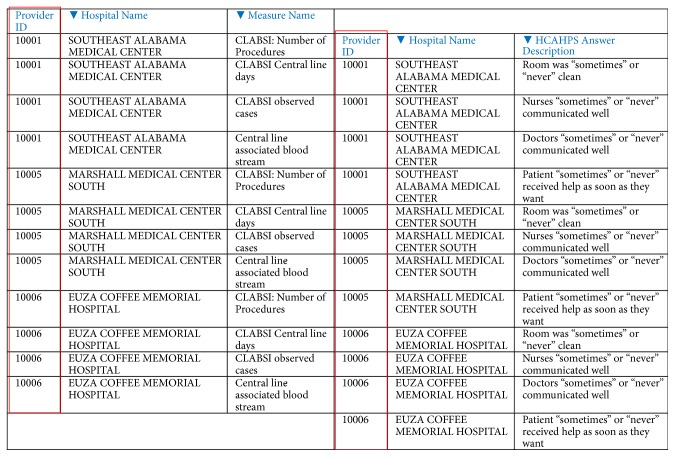
The two integrated datasets with the “Provider ID” attribute.

The next task is* data integration* of the two datasets. Besides the main attributes in the two datasets, another attribute (“Provider ID”) was also selected in both datasets. The “Provider ID” is a unique number that represented a hospital. It is important to note that a “Provider ID” represented the same hospital in both datasets. As a result, the integrated dataset consisted of 8 attributes for the 4,638 hospitals.  Figure 2 shows the two datasets with the attribute “Provider ID.” Thus, the two datasets were integrated on the basis of “Provider ID” attribute (in red rectangle), representing the same hospital. Dataset on the left is a part of NHSN: HAI dataset, while on the right it is a part of HCAHPS dataset.

### 4.3. Data Preparation

Data preparation is also called data preprocessing. In this phase, we take data identified in last step and prepare it for analysis by DM methods. Based on data understanding the data miner can verify the quality of collected data. Good quality of data will result in better learning and in turn more accurate predictions from the DM methods and vice versa. The good quality of data is evaluated in terms of data completeness, consistency, availability of required variables, and number of cases available. This step consumed most of the time (about 80% of the total time) and effort in the whole DM process. The major reasons behind this include incomplete and noisy data and unavailability of some required variables. Thus, it required* data cleaning* and* data transformation*. We explain them in the following subsections.

#### 4.3.1. Data Cleaning

**Figure 3:**
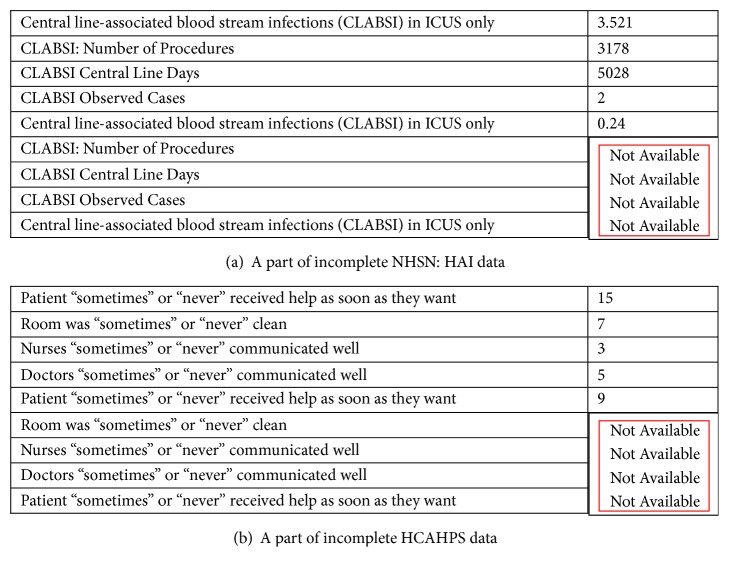


Data cleaning in DM refers to removing (or inferring or computing or correcting) incomplete/inconsistent and outlier values. Some of the attribute values for some hospitals were missing from NHSN: HAI as well as HCAHPS datasets, as shown in Figures 3(a) and 3(b), respectively. If data from the hospitals with incomplete attribute values is inputted to DM methods, it will negatively affect the prediction accuracy of the DM method. Therefore, we excluded hospitals that have incomplete data from our dataset. In Figures 3(a) and 3(b), the red rectangles indicate unavailable attributes of incomplete data.

**Figure 4:**
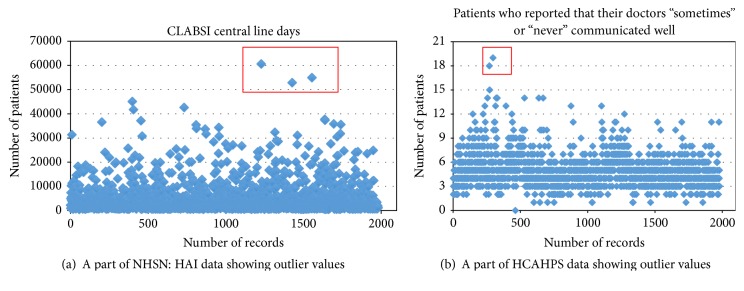


Another subtask in data cleaning was to ensure no outlier values in the dataset. We found some outlier values in data from both datasets. Figures 4(a) and 4(b) show some outlier values in two attributes “CLABSI: observed cases” and “patients who reported that their doctors sometimes or never communicated well,” respectively. DM models developed with outlier values yield very poor accuracy [59]. Therefore, we excluded hospitals that have outlier values from our experimental dataset,* during data preparation processes*; this is done using the visual inspection method [60]. For example, in Figures 4(a) and 4(b), the red rectangles indicate the outlier values excluded from the datasets.

#### 4.3.2. Data Transformation

Standardized infection ratio (SIR) is widely used metric by infection control units to measure infection rate [61]. This important attribute was missing in the collected dataset.

We computed SIR as a ratio of “CLABSI: observed cases” and “CLABSI: predicted cases” as shown in (1).

(1) $$\begin{array}{c} \text{SIR}=\frac{\left(\text{CLABSI Observed}\right)}{\left(\text{CLABSI Predicted}\right)}, \end{array}$$

where the value of the CLABSI predicted is

(2) CLABSI  Predicted=NHSN CLABSI rate×central line days1000.

Attributes in the selected dataset had different units and scales. Before processing them for DM, it is important to make them unit-free and scale them uniformly. To do so, we normalized each attribute as shown in (3), where *X*, *X*norm, *X*min, and *X*max represent current, normalized, minimum, and maximum values of an attribute, respectively:

(3) $$\begin{array}{c} Xnorm=\frac{\left(X-Xmin\right)}{\left(Xmax-Xmin\right)}. \end{array}$$

### 4.4. Model Construction

We modeled our prediction problem as a regression model in DM. RapidMiner tool [53] is used to develop prediction models using different DM methods. For evaluating our project,* the* DM* methods with higher prediction accuracy* were selected, among which are logistic regression, Naive Bayesian inference, multilayer perceptron, support vector machine, random forest, and AdaBoost. These algorithms are also chosen from the top ten DM algorithms [62]. A brief description of these DM algorithms is explained as shown in Table 1. While developing our models, we took special care to avoid overfitting [59] of the models. It is important to note that the model building process was an iterative process. The developed models were investigated for their error distribution to identify the segments where the models were less effective. Based on this investigation, model parameters were iteratively adjusted to improve model accuracy.

### 4.5. Testing and Evaluation

The developed models were tested and evaluated for their accuracy and generality. For this purpose, we split out the dataset into* training dataset* and* test dataset*. The* training dataset* was used for model construction and the* test dataset* was used for testing and evaluation purposes. We evaluated accuracy of our models using 10-fold standard cross validation, where the dataset was divided into 10 equal parts. Nine of these parts were used to develop our models and the 10th part was used for testing and evaluation.

**Figure 5:**
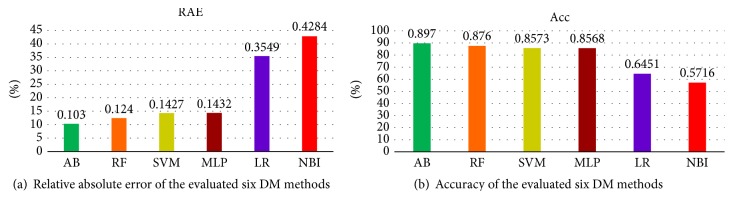


This process was repeated 10 times where a different part was used for testing and evaluation. The accuracy of the 10 iterations was averaged to show the accuracy of a model. The accuracy of a model was measured in terms of* absolute relative error (ARE)* computed as shown in (4), where *Ip* and *Ia* represent predicted and actual number of infections, respectively. Results are shown in Figures 5(a) and 5(b), respectively.

(4) Absolute Relative ErrorARE=Ip−IaIa,Accuracy=1−ARE.

**Figure 6:**
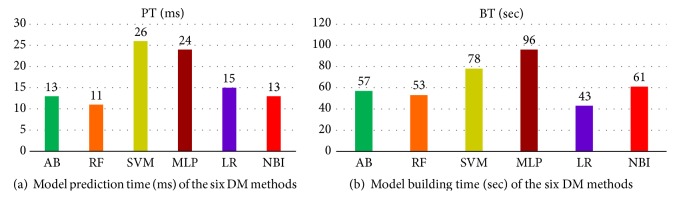


We found AdaBoost method as the best model. It resulted in the least error (10.3%). This achieved (89.7%) accuracy. We also evaluated the six selected DM models for their* speed* for model construction and* prediction*.  Figure 6(a) shows comparison of the six DM methods in terms of average CPU time taken to make predictions (in milliseconds), while Figure 6(b) shows comparison of the six DM methods in terms of average CPU time taken to build models (in seconds).

AdaBoost took the third smallest time in model building (57 seconds) and second smallest time in making predictions (13 ms). Although the random forest algorithm is faster than AdaBoost in model building as well as making predictions, yet the prediction accuracy of AdaBoost is higher than random forest. The speed of model building and predicting is not critical for prediction of CLABSI, so that AdaBoost is the best model based on its accuracy of predictions, since it is fast and easy to program.

### 4.6. Model Deployment

This is the last phase of CRISP-DM process in which the selected model (AdaBoost) will be deployed as software in different hospitals of Saudi Arabia. Initially, we are planning to deploy it in the clinical setup of King Abdulaziz University Hospital. At present, we are in the process of finalizing our software. The model deployment will be followed by necessary staff training for the use of the software. We believe that the developed software will help a lot in infection control as well as reducing the use of important resources.

**Figure 7:**
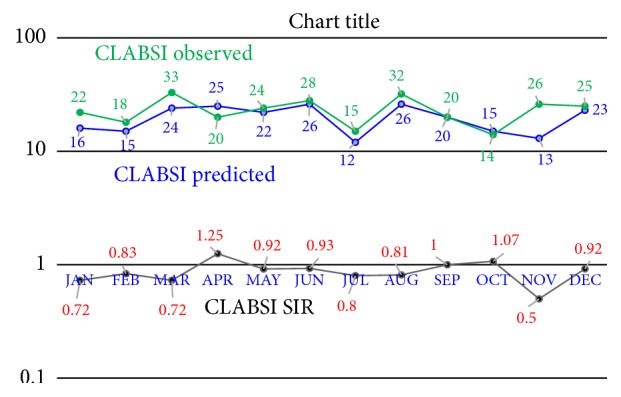
CLABSI predicted and observed patients and SIR.

We have implemented an experimental clinical surveillance program for detecting CLABSI.  Figure 7 shows some of the output results in the year 2016. The standard value of SIR is one. When SIR has value below one, it indicates better hospital safety and vice versa. Results in Figure 7 show that SIR has the highest value in April* (this was due to critical cases of some patients)*, while it has the lowest value in November. The high SIRs reflect a need for stronger CLABSI prevention efforts, while low SIRs reflect robust CLABSI prevention strategies.

## 5. Conclusion

Prediction of nosocomial infections is an important part of clinical surveillance programs to enable medical and healthcare practitioners take preventive actions in advance. The use of data mining (DM) proved to be very useful in the development of clinical surveillance programs, especially to predict the nosocomial infections. In this study, we begin with challenges in implementing a DM process for clinical surveillance program. The cross industry standard processes for DM are used to develop DM methods to predict central line-associated blood stream infections (CLABSIs). We used the dataset of healthcare-associated infections in US hospitals from* National Healthcare Safety Network* and integrated it with consumer survey data from* Hospital Consumer Assessment of Healthcare Providers and Systems*. Six DM methods are compared. The AdaBoost method can predict possibility of CLABSI with 89.7% accuracy. Thus, we can take preventive measures in advance to avoid the infections and in turn reduce length of patients' stay at hospital. Such predictive measures will save a lot of money and spare necessary resources for other patients.

## Figures and Tables

**Table 1:** The six data mining methods used.

No	DM Method	Description
1	AdaBoost (AB)	*AdaBoost* [40], short for “Adaptive Boosting,” is “a machine learning meta-algorithm. It is a powerful classification algorithm that has practical success with applications in a wide variety of fields. Boosting is an approach to machine learning based on the idea of creating a highly accurate prediction rule by combining many relatively weak and inaccurate rules.”

2	Random forest (RF)	*Random forest* [41] is “an ensemble learning method for classification and regression that operate by constructing a multitude of decision trees at training time and outputting the class that is the mode of the classes (classification) or mean prediction (regression) of the individual trees. Random decision forests correct for decision trees' habit of overfitting to their training set.”

3	Support vector machine (SVM)	The *SVM* [42] is used “to find the best classification function to distinguish between members of two classes in the training data. The metric for the concept of the ‘best' classification function can be realized geometrically. It is considered a good classifier because of its high generalization performance without the need to add a priori knowledge, even when the dimension of the input space is very high.”

4	Multilayer Perceptron (MLP)	The *MLP* [43, 44] is “a feedforward artificial neural network model that maps sets of input data onto a set of appropriate outputs. An MLP consists of multiple layers of nodes in a directed graph, with each layer fully connected to the next one.”

5	Logistic regression (LR)	*Logistic regression* [45] is “a statistical method for analyzing a dataset in which there are one or more independent variables that determine an outcome. The outcome is measured with a dichotomous variable (in which there are only two possible outcomes).”

6	Naive Bayesian inference (NBI)	*Naïve Bayesian inference* [46] is “a method of statistical inference in which Bayes' theorem is used to update the probability for a hypothesis as more evidence or information becomes available. Bayesian inference is an important technique in statistics and especially in mathematical statistics.”
